# HEALTH AND WELL-BEING AFTER DISPLACEMENT IN LATE LIFE AMONG ASIANS

**DOI:** 10.1093/geroni/igz038.708

**Published:** 2019-11-08

**Authors:** Wenjun Li, Allen Glicksman, Shantha Balaswamy

**Affiliations:** 1 University of Massachusetts Medical School, Worcester, Massachusetts, United States; 2 Philadelphia Corporation for Aging, Philadelphia, Pennsylvania, United States; 3 The Ohio State University, Columbus, Ohio, United States

## Abstract

Displacement in late life may cause social and health issues. Displaced older adults may experience language and cultural barriers, loss of social networks, difficulties in navigation in new physical and social environments, limited options in transportation and mobility, delayed access to health care, and thus sudden or gradual loss of their autonomy, and increasing dependence on their adult children. These issues are known but not well understood, and effective interventions are yet to do developed. This symposium brings together four studies that address several critical social and health issues among late-life Asian immigrants. Dr. Inoue discusses use of person-centered care to reduce social isolation and loneliness among old and ill Asian immigrants in long term care settings. Ms. Ring examines racial differences in navigation and access to long term aging services and social supports (LTSS), and evaluates the use of Social Interaction Modeling to help connect limited English-speaking minorities in need to the formal LTSS system. Dr. Torres from Sweden provides a systematic review of research into racial/ethnic differences in health and social care, and from an international perspective, advocates a research agenda that is both diversity-astute and injustice-aware. In settings where self-report data are likely unreliable, Dr. Li demonstrates an innovative method for objectively measuring spatiotemporal patterns of physical and social activities and use of neighborhood resources among non-English speaking late-life immigrants. Together these studies demonstrate that existing methods can be adapted and new methods can be created to answer important health and social issues among late-life Asian immigrants.

